# Risk Factors for Mortality in Severely Ill Children Admitted to a Tertiary Referral Hospital in Malawi

**DOI:** 10.4269/ajtmh.19-0127

**Published:** 2019-07-08

**Authors:** Fatsani Ngwalangwa, Chikondi H. A. Phiri, Queen Dube, Josephine Langton, Helena Hildenwall, Tim Baker

**Affiliations:** 1 Department of Paediatrics, College of Medicine, University of Malawi, Blantyre, Malawi;; 2 Queen Elizabeth Central Hospital, Blantyre, Malawi;; 3 Department of Public Health Sciences, Karolinska Institutet, Global Health–Health System and Policy Research Group, Stockholm, Sweden;; 4 Astrid Lindgren Children’s Hospital, Karolinska University Hospital, Stockholm, Sweden

## Abstract

In low-resource settings, many children are severely ill at arrival to hospital. The risk factors for mortality among such ill children are not well-known. Understanding which of these patients are at the highest risk could assist in the allocation of limited resources to where they are most needed. A cohort study of severely ill children treated in the resuscitation room of the pediatric emergency department at Queen Elizabeth Central Hospital in Malawi was conducted over a 6-month period in 2017. Data on signs and symptoms, vital signs, blood glucose levels, and nutritional status were collected and linked with in-hospital mortality data. The factors associated with in-hospital mortality were analyzed using multivariable logistic regression. Data for 1,359 patients were analyzed and 118 (8.7%) patients died. The following factors were associated with mortality: presence of any severely deranged vital sign, unadjusted odds ratio (UOR) 2.6 (95% CI 1.7–4.0) and adjusted odds ratio (AOR) 3.2 (95% CI 2.0–5.0); severe dehydration, UOR 2.6 (1.4–5.1) and AOR 2.8 (1.3–6.0); hypoglycemia glycemia (< 5 mmol/L), UOR 3.6 (2.2–5.8) and AOR 2.7 (1.6–4.7); and severe acute malnutrition, UOR 5.8 (3.5–9.6) and AOR 5.7 (3.3–10.0). This study suggests that among severely sick children, increased attention should be given to those with hypo/low glycemia, deranged vital signs, malnutrition, and severe dehydration to avert mortality among these high-risk patients.

## INTRODUCTION

In 2016, 5.6 million children younger than 5 years died worldwide.^1^ More than half of the deaths were due to conditions that could be prevented or treated with simple and affordable interventions.^2^ For those that reached the hospital, approximately half of the deaths occurred within the first 24 hours of admission suggesting a large burden of acute, severe illness at presentation to hospital.^3^

The WHO developed the Emergency Triage Assessment and Treatment (ETAT) guidelines in 2005, as a tool aiming to improve the care for acutely ill children in hospitals.^4^ In these guidelines, children with emergency features are identified on arrival to the hospital for immediate stabilization and treatment. The introduction of ETAT and restructuring of the admission process reduced in-hospital mortality from 18% to 8% in a Malawian referral hospital.^5^ Similar tools for identifying children at high risk of deterioration or mortality have been developed in other settings, such as the Pediatric Risk of Mortality^6^ score, the Pediatric Index of Mortality (PIM),^7^ and the Pediatric Early Warning Score (PEWS).^8^

However, these tools have some weakness. Pediatric Risk of Mortality and PIM were developed in high-resource settings and involve laboratory tests, which are not available in low-resource settings. Pediatric Early Warning Score adds up bed-side vital signs into compound scores, an approach which may be time-consuming and impractical for use in settings with low levels of human resources.^9^ Emergency Triage Assessment and Treatment does not identify individuals at the highest risk of mortality within the emergency triage category, and ETAT implementation alone is not always sufficient to address mortality among pediatric in-patients.^10^

Understanding which simple factors indicate the highest risk of death among severely ill children could help identify the sickest children and enable efficient allocation of resources. In this study in a tertiary hospital in Malawi, we investigated the factors at arrival to the hospital that are associated with mortality among children already triaged as severely ill.

## METHODS

This cohort study enrolled patients prospectively from the pediatric accident and emergency department in Queen Elizabeth Central Hospital (QECH), a tertiary referral hospital in the southern region of Malawi. Annually, the pediatric department serves 100,000 children and admits 24,000. The ETAT was introduced at QECH in 2001,^5^ and severely ill children with a WHO emergency sign are identified at triage and referred into the resuscitation room for immediate treatment and stabilization before admission and continued management. In the resuscitation room, children presenting with severe respiratory distress or low oxygen saturation receive oxygen therapy. Children with shock or severe dehydration have intravenous or intraosseous access secured and receive intravenous crystalloid; the volume and rate are determined by the clinical presentation. The hospital has a six-bedded pediatric intensive care unit (PICU) with the potential for providing mechanical ventilation. However, because of limited capacity, there are strict guidelines as to which patients qualify for PICU admission such that only a very small proportion of severely sick children are admitted. We included all children aged up to 17 years who were triaged as severely ill to the resuscitation room between first of April and 30th of September 2017. The primary outcome was in-hospital mortality and the secondary outcome was 24-hour mortality.

All children had clinical and demographic data recorded on hospital documentation. A standardized electronic Case Reporting Form was used to collect the data. Pediatric in-hospital deaths were recorded and linked to the hospital data, and the database was anonymized for analysis.

The variables of interest which were predefined by the researchers for clinical plausibility were 1) age; 2) gender; 3) any severely deranged vital sign; 4) an airway or breathing emergency sign; 5) shock; 6) severe dehydration; 7) coma or convulsion; 8) blood sugar level (normoglycemia [≥ 5–10 mmol/L], hypo/low glycemia [< 5 mmol/L], and hyperglycemia [> 10 mmol/L])^11^; 9) referral from another health facility; 10) prolonged fasting (> 8 hours of reported fasting); and 11) severe acute malnutrition (as diagnosed by the clinician.)

**Figure 1.**
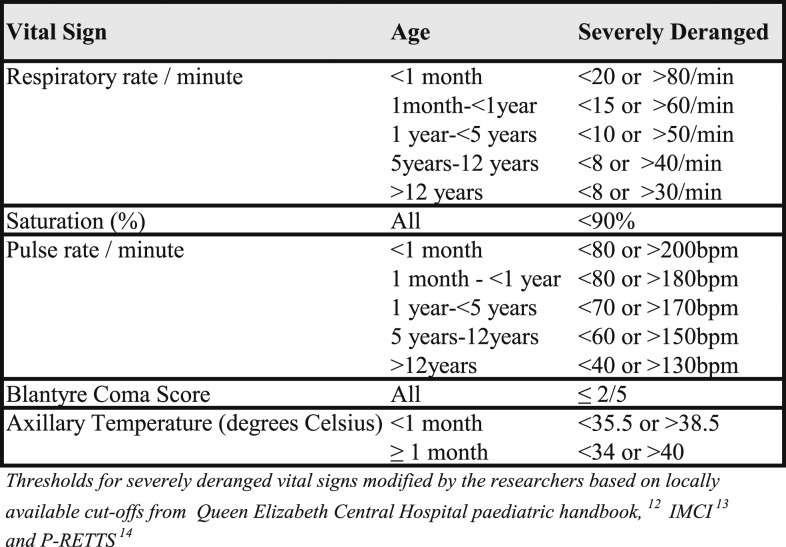
Classification of severely deranged vital signs by age.

Vital signs were analyzed as binary (severely deranged or not severely deranged) and the age-specific cutoffs for severe derangements were defined a priori by the authors (Figure 1) based on locally available thresholds^12^ and related guidelines for the Integrated Management of Childhood Illness and Pediatric Rapid Emergency Triage and Treatment tool (P-RETTS).^13,14^ The emergency signs (obstructed or absent breathing, severe respiratory distress, shock, severe dehydration, coma, and convulsion) were defined as per WHO ETAT guidelines.^15^ Clinical concern was defined according to the triage team’s assessment and included any child who did not fulfill the criteria of an emergency sign but whose condition was life-threatening. Severe acute malnutrition was based on the clinical judgment of the admitting team (based on clinical signs: the presence of visible severe wasting, bilateral pitting edema, and mid-upper arm circumference < 11.5 cm).

Blood sugar levels were defined as hypo/low glycemia if less than 5 mmol/L as several studies have shown increased mortality in low glycemia (2.5–5 mmol/L) and hypoglycemia (< 2.5 mmol/L).^16,17^ A subanalysis analyzed low glycemia and hypoglycemia separately.

The required sample size was calculated using 10 events per predefined variable as suggested by Hosmer et al.^18^ and Peduzzi et al.^19^ As 11 covariates were included, 110 events (deaths) were required. Queen Elizabeth central hospital has an average of 20 deaths per month for children treated in the resuscitation room (unpublished data from the department), so data collected in a 6-month period were required.

The data were analyzed using Stata version 15 (Statacorp, College Station, TX). Proportions and means were used where appropriate. Univariate and multivariable logistic regression, including all the defined variables were used to estimate odds ratios with a 95% confidence interval and significance level of 0.05. Data imputation was used for all missing observations assuming that all missing data were normal. Sensitivity analysis was carried out to investigate the robustness of the imputation.

The study was approved by the University of Malawi College of Medicine Research Ethics Committee (P.08/16/2007).

## RESULTS

A total of 1,365 patients were treated in the resuscitation room during the 6-month period. Data were not recorded in six (0.4%) patients: five died just after arrival and one was admitted to the ward without recorded data. Thus, 1,359 patients were included in the study. The mean age was 4 years (range 2 days–17 years) and 794 (58.4%) were males. One hundred eighteen (8.7%) patients died in hospital. Severe respiratory distress was the most common WHO emergency sign present in 563 (41.4%) patients. One hundred thirty-seven (10.1%) of the children had hypo/low glycemia and 211 (15.5%) had hyperglycemia. The majority (79.3%) were referred to QECH from another medical facility. The characteristics of the children who died and those who survived are presented in Table 1.

**Table 1:** Baseline characteristics of the study participants

Variable	All (*N* = 1,359) *n* (%)	Survivors (*N* = 1,241) *n* (%)	Died (*N* = 118) *n* (%)
Age (years)			
0 < 1	417 (30.7)	375 (30.2)	42 (35.1)
1 < 5	570 (41.9)	530 (42.7)	40 (33.6)
≥ 5	372 (27.4)	336 (27.1)	36 (31.1)
Gender			
Female	565 (41.5)	513 (41.3)	52 (44.1)
Presenting emergency sign*			
Obstructed breathing	13 (1.0)	8 (0.6)	5 (4.2)
Central cyanosis	13 (1.0)	10 (0.8)	3 (2.6)
Severe respiratory distress	563 (41.4)	527 (42.5)	36 (30.5)
Shock	20 (1.5)	16 (1.3)	4 (3.4)
Coma	100 (7.4)	86 (6.9)	14 (11.9)
Convulsions	164 (12.1)	151 (12.2)	13 (11.1)
Severe dehydration	63 (4.6)	51 (4.1)	12 (10.2)
Clinical concern†	289 (21.3)	258 (20.8)	31 (26.5)
Other	88 (6.5)	79 (6.4)	9 (7.6)
None	73 (5.4)	67 (5.4)	6 (5.1)
Severe acute malnutrition‡	87 (6.4)	60 (4.8)	27 (22.9)
Blood sugar level (mmol)			
Normoglycemia (≥ 5 to < 10)	1,011 (74.4)	941 (75.8)	70 (59.3)
Hypo/low glycemia (< 5)	137 (10.1)	108 (8.7)	29 (24.6)
Hyperglycemia (≥ 10)	211 (15.5)	192 (15.5)	19 (16.1)
Any severely deranged vital sign§	672 (49.5)	589 (47.5)	83 (70.3)
Referred from other facility	1,077 (79.3)	977 (78.7)	100 (84.8)
Reported fasting hours			
< 8 hours	982 (72.3)	902 (72.7)	80 (67.8)
≥ 8 hours	377 (27.7)	339 (27.3)	38 (32.2)

* As some patients presented with more than one emergency sign, the total percentage may be greater than 100.

† The patients did not have any emergency sign, but the clinician was concerned that the patient was very sick with a risk of acute deterioration.

‡ Malnutrition as diagnosed by the clinician.

§ Any severely deranged respiratory rate, heart rate, temperature, oxygen saturation, or Blantyre coma score.

The factors associated with mortality were any severely deranged vital sign, unadjusted odds ratio (UOR) 2.6 (95% CI 1.7–4.0) and adjusted odds ratio (AOR) 3.2 (95% CI 2.0–5.0); the WHO emergency sign of severe dehydration, UOR 2.6 (1.4–5.1) and AOR 2.8 (1.3–6.0); hypo/low glycemia, UOR 3.6 (2.2–5.8) and AOR 2.7 (1.6–4.7); and severe acute malnutrition, UOR 5.8 (3.5–9.6) and AOR 5.7 (95% CI 3.3–10.0). When we defined hypoglycemia and low glycemia as separate categories, low glycemia, UOR 3.6 (2.1–6.1), and hypoglycemia, UOR 3.7 (1.6–8.4), were both associated with in-hospital mortality; hyperglycemia did not show an association. The other factors age, gender, presence of the WHO emergency signs for airway, breathing, coma or convulsions did not show an association with mortality. Of the 118 children who died, 39 (33%) died within the first 24 hours of admission. The following factors were associated with 24-hour mortality: any severely deranged vital sign, UOR 12.9 (4.0–42.0) and AOR 15.0 (4.5–50.3); hypo/low glycemia UOR 3.2 (1.4–7.1) and AOR 3.0 (1.2–7.6); and severe acute malnutrition, UOR 4.1 (1.8–9.1) and AOR 3.1 (1.3–7.6). The rest of the factors were not associated with 24-hour mortality (Table 3).

**Table 2:** Factors associated with in-hospital mortality in severely ill children

Variable	Unadjusted odds ratio	95% CI	*P* Value	Adjusted odds ratio*	95% CI	*P* Value
Age (Ref > 5 years)						
< 1 year	1.0	0.7–1.7	0.853	1.1	0.6–1.9	0.764
≥ 1 year to < 5 years	0.7	0.4–1.1	0.113	0.6	0.3–0.9	0.040
Gender (Ref male)						
Female	1.1	0.8–1.6	0.565	1.2	0.8–1.9	0.450
Any severely deranged vital sign†	2.6	1.7–4.0	< 0.001	3.2	2.0–5.0	< 0.001
WHO emergency sign						
Airway/breathing signs‡	0.7	0.5–1.1	0.122	0.6	0.4–1.1	0.081
Shock	2.7	0.9–8.2	0.082	1.3	0.4–4.7	0.645
Severe dehydration	2.6	1.4–5.1	0.004	2.8	1.3–6.0	0.008
Coma/convulsion sign§	1.2	0.7–1.8	0.554	0.9	0.5–1.8	0.99
Blood sugar level (mmol/L) (Ref normoglycemia)						
Hypo/low glycemia (< 5)	3.6	2.2–5.8	< 0.001	2.7	1.6–4.7	< 0.001
Hyperglycemia (> 10)	1.3	0.8–2.3	0.291	1.3	0.7–2.2	0.404
Referred from another facility	1.5	0.9–2.5	0.126	1.6	0.9–2.9	0.081
Fasting time (Ref < 8 hours)						
≥ 8 hours	1.3	0.8–1.9	0.258	1.0	0.6–1.6	0.989
Severe acute malnutrition	5.8	3.5–9.6	< 0.001	5.7	3.3–10.0	< 0.001

Ref = reference group.

* Adjusted for all the other variables in the table.

† Presence of any severely deranged respiratory rate, heart rate, temperature, oxygen saturation, or Blantyre coma score.

‡ Presented with either airway obstruction or severe respiratory distress WHO emergency sign.^15^

§ Presented with either coma or convulsion WHO emergency sign.^15^

**Table 3:** Factors associated with 24-hour mortality in severely ill children

Variable	Unadjusted odds ratio	95% CI	*P* Value	Adjusted odds ratio*	95% CI	*P* value
Age (Ref ≥ 5 years)						
< 1 year	1.2	0.5–2.7	0.721	0.9	0.4–2.6	0.906
≥ 1 year to < 5 years	1.0	0.5–2.3	0.971	0.9	0.4–2.2	0.806
Gender (Ref male)						
Female	0.9	0.5–1.7	0.689	0.8	0.4–1.6	0.579
Any severely deranged vital sign†	12.9	4.0–42.0	< 0.001	15.0	4.5–50.3	< 0.001
WHO emergency sign						
Airway breathing sign‡	1.4	0.7–2.6	0.569	0.8	0.4–1.7	0.569
Shock	1.8	0.2–13.8	0.570	1.0	0.1–9.4	0.974
Severe dehydration	1.8	0.5–5.9	0.334	2.0	0.5–7.9	0.314
Coma/convulsion sign§	0.4	0.1–1.2	0.095	0.2	0.1–9.0	0.033
Blood sugar level (mmol/L) (Ref normoglycemia)						
Hypo/low glycemia (< 5)	3.2	1.4–7.1	0.004	3.0	1.2–7.6	0.017
Hyperglycemia (> 10)	1.7	0.8–4.0	0.185	1.8	0.8–4.2	0.185
Referred from another facility	1.2	0.5–2.8	0.665	1.1	0.5–2.8	0.759
Fasting time (Ref < 8 hours)						
≥ 8 hours	1.0	0.5–2.1	0.945	0.9	0.4–2.0	0.794
Severe acute malnutrition	4.1	1.8–9.1	0.001	3.1	1.3–7.6	0.014

Ref = reference group.

* Adjusted for all the other variables in the table.

† Presence of any severely deranged respiratory rate, heart rate, temperature, oxygen saturation or Blantyre coma score.

‡ Presented with either airway obstruction and/or severe respiratory distress WHO emergency sign.^15^

§ Presented with either coma and/or convulsion WHO emergency sign.^15^

Normal values were imputed for the 1.4% of observations that were missing. There were no substantial differences in the results when sensitivity analyses were carried out using single abnormal imputation or complete case deletion.

## DISCUSSION

We found that any severely deranged vital sign, hypo/low glycemia, severe acute malnutrition, and the WHO emergency sign of severe dehydration were risk factors for in-hospital mortality among children triaged as severely ill at arrival to a tertiary hospital in Malawi. These factors were also associated with 24-hour mortality except for severe dehydration.

The presence of a severely deranged vital sign appears to indicate an increased risk of dying in the first 24 hours and during the hospital stay among children who were already triaged as high risk. This emphasizes the importance of early identification of children with severely deranged vital signs right at admission which could aid early stabilization and prioritization of resources. The association between severely deranged vital signs and mortality concurs with studies carried out in other settings^20–22^ and is the rationale behind the use of vital signs in triage systems such as P-RETTS^23^ and PEWS.^24^ Severely deranged vital signs have shown to precede negative outcomes such as ICU transfer,^25^ life-threatening events in the hospital,^26^ cardiac arrest,^27^ and mortality.^28^ In Malawi, the Inpatient Triage And Treatment score,^22^ which was based on four vital signs (respiratory rate, pulse rate, temperature, and oxygen saturation) was able to identify children at high risk of death. However, this was carried out on an unselected population of admitted children and was based on a compound score which may be complicated to use in low-resource settings.^21^ Presence of any severely deranged vitals in a severely ill child calls for prioritization and immediate stabilization.

Hypo/low glycemia could be a marker of disease severity and failing body physiology and/or may indicate poor feeding among very sick children.^29^ Alternatively, it may be a causative factor itself for mortality.^30^ Presence of hypo/low glycemia in severely ill child should prompt prioritization of the child, early glucose administration and prevention of further falling of the blood sugar to reduce mortality. The association between low blood glucose and in-hospital mortality as well as 24-hour mortality has been reported from several studies carried out in sub-Saharan Africa.^16,17,31,32^ However, most of these studies did not focus on severely ill children only. Studies have questioned the current cutoff for glucose administration as increased mortality has been seen in children with low glycemia just like in this study.^16,17^ WHO uses a cutoff of < 2.5 mmol/L to administer glucose treatment to children^33^ leaving out children with low glycemia who might equally be at risk. Currently, a study is being conducted to look at the impact of administering blood glucose in children with low glycemia on mortality.^34^

Severe acute malnutrition was shown to be a risk factor for in-hospital death and for death within the first 24 hours. Severe malnutrition complicates disease presentation^35^ and is usually associated with multiple comorbidities.^36^ The presence of a severe illness in a severely malnourished child may indicate that the child is at high risk of early mortality, and prioritization and stabilization of severely malnourished children need to be carried out right at admission. Severe acute malnutrition has been widely studied and is recognized as a risk factor for death and for 24-hour mortality in other studies.^37–40^

Severe dehydration among severely ill children appears to be a particularly dangerous emergency sign. The increased risk of mortality may be due to illness severity, challenges with optimizing fluid management, or difficulties in differentiating septic hypovolemia and dehydration because the two may have similar clinical presentations, but the fluid management is different. Fluid boluses in septic patients have been shown to be detrimental in the recent Fluid Expansion And Supportive Therapy study (FEAST) study,^41^ hence, the recommendation of fluid restriction by WHO in septic patients. Although this does not include severely dehydrated patients, it may have led to changed fluid management even in dehydrated children.

The study had some limitations. It was conducted in a teaching hospital, which is relatively well equipped but still resource limited and may not reflect what happens in other low-income country hospitals. The study was conducted for a period of 6 months and may not capture seasonal variability. The data were collected during an ongoing randomized controlled trial investigating the impact of providing dextrose to patients with low glycemia, and this may have affected the risk of mortality for those low-glycemic children who had been randomized to receive additional dextrose, potentially biasing the findings for this group. The strengths of the study are that although several studies have been carried out looking at the risk factors of mortality in children, this is the only study that looks at the admission risk factors of mortality among children already triaged as severely ill. This study included all severely ill children irrespective of underlying diseases and included children across all pediatric age groups.

In conclusion, the study identified severely deranged vital signs, hypo/low glycemia, severe malnutrition, and the WHO emergency sign of severe dehydration as risk factors for mortality among children already triaged as severely ill. These are routinely measured in all severely ill children and special attention should be given to children presenting with these factors to avert in-hospital mortality.
